# Milk from Sewage Farms

**Published:** 1896-10

**Authors:** 


### Milk from Sewage Farms.

If a cow is fed on turnips, within twenty-four hours her milk will taste of turnips, and if butter is churned from the cream the butter will taste, too. The intensity of the turnip flavor is the measure of the quantity of turnips taken. In like manner, if pigs are fed on horseflesh, as they often are, their bacon will taste of the horseflesh; if they are fed on fish the bacon has a fishy taste. The same is true of hens and their eggs. Feed hens on decaying animal matter, which they will eat greedily, and both their eggs and flesh will be most unpleasant and unwholesome eating. In the case of ducks the facts are much more striking. Ducks are very unclean feeders. Give them an abundance of garbage, and they will refuse corn and similar food. Their flesh is then most pungent to the taste, and in many people is so potently poisoning as to produce diarrhœa. Animals fed on sewage farms, under certain conditions, are liable to have their flesh and secretions changed in character by the sewage-produced herbs and grasses upon which they feed. If the sewage on a given farm be so managed that no more of it be put into the soil than any given crop can adequately deal with, then the crop will be sweet and natural, and the cattle or other animals fed on it will be sweet and natural, too. But if the soil be gorged to repletion with sewage, then the crop will be surcharged with sewage elements, and is unfit for food, and the meat and milk of animals fed on such a crop will be like the crop, and very unpleasant to the taste as well as dangerous to health. It is in the last resort all a question of the intelligence and conscience of the managers of sewage farms.—*Ind. Lancet.*

